# Does Co-Morbid Obsessive–Compulsive Disorder Modify the Abnormal Language Processing in Schizophrenia Patients? An fMRI Study

**DOI:** 10.3389/fnhum.2014.00560

**Published:** 2014-07-29

**Authors:** Maya Bleich-Cohen, Michael Poyurovsky, Talma Hendler, Ronit Weizman, Haggai Sharon

**Affiliations:** ^1^Functional Brain Center, Wohl Institute for Advanced Imaging, Tel Aviv Sourasky Medical Center, Tel Aviv, Israel; ^2^Research Unit, Tirat Carmel Mental Health Center, Tirat Carmel, Israel; ^3^Rappaport Faculty of Medicine, Technion – Israel Institute of Technology, Haifa, Israel; ^4^Sackler Faculty of Medicine, Tel Aviv University, Tel Aviv, Israel; ^5^School of Psychological Sciences, Tel Aviv University, Tel Aviv, Israel; ^6^Sagol School of Neuroscience, Tel Aviv University, Tel Aviv, Israel; ^7^Clinical Psychopharmacology Unit, Tel Aviv Community Mental Health Center, Tel Aviv, Israel

**Keywords:** language, fMRI, inferior frontal gyrus, schizo-obsessive, schizophrenia, OCD

## Abstract

**Background:** Impaired language processing is one of the most replicated findings in functional brain studies of schizophrenia (SCH). This is demonstrated by reduced activations in left prefrontal language areas (i.e., BA44/45, the inferior frontal gyrus, IFG) presented as decreased language lateralization. This finding was documented both in chronic as well as in first-episode SCH patients, arguing for a neurobiological marker for SCH. In a previous study, we demonstrated the specificity of this finding to SCH patients when compared to obsessive–compulsive disorder (OCD) patients in whom language processing was similar to healthy controls. Since a sizable proportion of SCH patients also meet DSM-IV criteria for OCD, we further sought to elucidate whether OCD attenuates abnormal prefrontal language lateralization in this unique group of schizo-obsessive patients compared to their non-OCD-SCH counterparts.

**Methods:** We used functional magnetic resonance imaging (fMRI) to investigate regional activation and language lateralization in the left and right IFG and inter-hemispheric functional connectivity (FC) during a language task of auditory verb generation in 14 SCH patients with OCD, compared to 17 SCH patients without OCD, 13 OCD patients and 14 healthy controls.

**Results:** No between-group differences were found in the behavioral measurements of word generation. However, while OCD patients were indistinguishable from healthy volunteers, a similarly reduced lateralization in the IFG and diminished inter-hemispheric FC was noted in the two SCH groups with and without OCD.

**Conclusion:** The co-occurrence of OCD in SCH does not attenuate abnormal processing of language as reflected by regional IFG activity and FC. These results further support the notion that these language processing abnormalities are characteristic of SCH and that SCH–OCD combined psychopathology is more akin to SCH than to OCD.

## Introduction

The prefrontal cortex (PFC) is a large and complex structure containing a number of sub-regions, each involved in mediating important high-order mental processes such as decision making, emotion regulation, selective attention, language processing, social cognition and many others. Every human psychopathology involves dysfunction in at least one subdivision of the PFC. For instance, obsessive–compulsive disorder (OCD) patients display PFC dysfunction mainly (but not only) in the orbitofrontal cortex (OFC) (Rauch et al., 1994). On the other hand, Schizophrenia (SCH) patients exhibit abnormalities in every sub-region of the PFC and every functional domain associated with it, such as the OFC in emotion regulation and social cognition (Bleich-Cohen et al., 2009b), dorso-lateral PFC in working memory (Bleich-Cohen et al., 2014), and inferior frontal gyrus (IFG) in language control (Bleich-Cohen et al., 2012). Thus, it is especially interesting to probe PFC activity in the context of psychiatric co-morbidity. Among the many questions that can be raised are – will the pattern of PFC dysfunction be a cumulative one? Will it result in a different pattern of abnormality altogether? Can one pattern of dysfunction in a specific disorder offer a protective mechanism in the context of co-morbidity with another?

There is compelling evidence indicating that obsessive–compulsive symptoms (OCS) are common and clinically significant phenomena in SCH. When SCH patients also meet the criteria for OCD, the combined co-morbidity is termed “Schizo-obsessive disorder.” Such co-morbidity seems to modify the course, psychopathological features, and treatment response in SCH patients, and exert a modifying effect on the severity of the symptoms of SCH, with several groups reporting lower severity of some positive and negative symptoms (Poyurovsky et al., 1999), and others, no difference or even more severe positive as well as negative symptoms in chronic schizo-obsessive patients (Fenton and McGlashan, 1986; Lysaker et al., 2000). A recent meta-analysis (Cunill et al., 2009) found that while OCS in SCH patients were associated with more negative and more positive symptoms, such a relation was not found in frank OCD co-morbidity in SCH patients.

Of the prefrontal control systems, one of the most extensively studied, in both health and disease, is that of language processing, which mainly relies on the functional activity pattern of the left IFG and its inter-hemispheric connectivity. The domain of language can therefore serve to evaluate a key aspect of PFC function and connectivity across different brain disorders, as well as in complex psychiatric syndromes. A repeated brain imaging finding in SCH patients is decreased asymmetry of language-related activation, mostly confined to Broca’s area located in the left IFG (BA 44,45) (Sommer et al., 2001, 2003; Bleich-Cohen et al., 2009a). The relation of this abnormal inter-hemispheric functional organization to the brain pathology of SCH is suggested by its occurrence in unmedicated SCH patients (Weiss et al., 2006) and in first episode of psychosis among SCH patients (Bleich-Cohen et al., 2009a). van Veelen et al. (2011) recently reported reduced language lateralization in the IFG at the onset of SCH, before medical treatment is initiated. Altogether, these findings argue for reduced language-related brain asymmetry as an early neural marker of SCH.

Behavioral data concerning language abilities in the case of OCD patients are conflicting. A number of behavioral studies show significant impairments in the fluency letter task in OCD patients when compared to controls (Schmidtke et al., 1998; Moritz et al., 2001, 2002), while others claim no significant between-group differences (Martinot et al., 1990; Basso et al., 2001). In contrast to SCH patients, there is no evidence for decreased language lateralization in OCD subjects. In a previous work by our group (Bleich-Cohen et al., 2012), we demonstrated the specificity of reduced language lateralization in SCH patients compared to OCD that were similar to healthy controls. Hence, it seems that even if OCD patients may suffer from certain impairments in verbal fluency tasks, there is still no evidence for decreased language brain lateralization. Moreover, Lee et al. (2009) have recently demonstrated that OCD may have a protective effect on some cognitive functions, with OCD–SCH patients performing better at the Stroop-interference and verbal fluency tests, which are highly dependent on executive function.

Therefore, it is interesting to examine what, if any, are the language processing abnormalities that can be found in patients who meet criteria for both disorders. The aim of the present functional MRI study was to examine language lateralization in a well-defined group of schizo-obsessive patients when compared to SCH patients without OCD, OCD patients without other co-morbidities and healthy controls. Following our previous finding of abnormal language lateralization in SCH but not in OCD, we wished to evaluate whether OCD can act as a protective mechanism in the domain of language processing in the left IFG in the schizo-obsessive group when compared to pure SCH.

The present study can contribute to a better understanding of the function of the PFC in complex psychopathology, as well as to better identification and pathophysiological characterization of this complex combined disorder.

## Materials and Methods

### Study population

Four groups were evaluated in this study. The study group included 13 inpatients (10M, 3F; age: 19–32 years) from Tirat Hacarmel Mental Health Center who met DSM-IV criteria for both SCH and OCD. The comparison groups included 17 SCH patients (12M, 5F; age: 20–34 years), 13 OCD patients (10M, 3F; age: 19–31 years), and 18 healthy volunteers (control groups) (12M, 6F; age: 20–35 years). Two SCH patients with OCD, four SCH patients, and four healthy controls were excluded from the final analyses due to severe head movements and two SCH patients due to intolerance to the functional magnetic resonance imaging (fMRI) procedure. After excluding the layouts, 11 SCH patients with OCD, 13 OCD patients, 13 SCH patients, and 14 healthy controls were included in the analyses. All participants were right-handed native Hebrew speakers. The SCH patients were inpatients at the Tirat Hacarmel Mental Health Center. The same psychiatrist verified the diagnosis according to the Structured Clinical Interview of DSM-IV (First et al., 1994). Exclusion criteria included other psychiatric disorder, acute physical illness, pregnancy or chronic substance abuse. Prior to imaging, severity and content of OCS were evaluated using the Yale–Brown Obsessive–Compulsive Scale [Y-BOCS (Goodman et al., 1989)]. Inclusion criteria for the schizo-obsessive group were: presence of typical OCS that is time-consuming (≥1 h per day), distressful obsessions, and/or compulsions that significantly interfered with patients’ functioning (ascertained by the checklist incorporated into the Y-BOCS). The duration criteria for OCS were set at ≥6 months. Severity of SCH symptoms was assessed with the Schedule for the Assessment of Positive Symptoms [SAPS (Andreasen, 1984)] and the Schedule for the Assessment of Negative Symptoms [SANS (Andreasen, 1983)]. Patients with affective and organic mental disorders or drug- or alcohol-induced psychoses were excluded. Medical and neurological illnesses were ruled out by physical and neurological examinations, routine laboratory investigation, reports from patients’ treating physicians and medical records. The study was approved by the Institutional Review Boards of both Tirat Carmel Mental Health Center and Tel Aviv Sourasky Medical Center. All participants gave written informed consent after receiving a full explanation of the study protocol.

### fMRI paradigm

While being scanned, the subjects participated in an auditory language task or listened to periods of music, interspersed with periods of no stimuli (rest). All subjects prior to entering the fMRI phase of the investigation underwent a preparatory session in which adequate compliance was documented and assured. Patients who for whatever reason demonstrated lack of complete understanding and compliance were not included in the study. The language task was composed of 18 spoken Hebrew words presented through headphones during three 18-s periods. Each block consisted of six different words, at a rate of 1/3 Hz. There were 24 s of no stimuli at the beginning and at the end of the paradigm: although MRI scanning is inherently accompanied by noise, the noise is constant and monotonous, whereupon “no stimuli” can be considered as “rest.” Each verbal period was alternated with three 18-s periods of classical music and five 9-s periods of rest. Two more rest periods of 30 s appeared in the beginning and ending of the experiment. Only one version of the experiment was used (the order of the music and verb generation tasks was not randomized between the participants). The verbal and musical periods were recorded separately and organized in a block paradigm to be presented by the GoldWave program (5.1.2600.2180; Microsoft Windows). The words were three- to five-letter nouns that described commonly used objects, such as a brush and a table. During the language condition, the subjects were instructed to think of (but not utter) a verb that best described what they could do with the object (noun) that was named. For example, when “cup” was heard, they could think of “drinking.” Each verbal period was terminated with an audible ring. The music condition consisted of an instrumental piece of classical music composed by Mozart. During the periods of music, the participants were instructed to listen passively to the music. They were told to do nothing during the rest condition.

### Outside the magnet

#### Behavioral testing

All participants were tested for performance on the same task by recording accuracy and reaction time of spoken responses. Verbal responses were recorded by Presentation software (Neurobehavioral Systems, Inc., 2003). Reaction times and accuracy scores were analyzed using two-tailed, unpaired, Student’s *t*-test.

#### Brain scanning

Imaging was performed using a 1.5 T GE Sigma Horizon LX 8.25 echo speed scanner (Milwaukee, WI, USA) with a resonant gradient echoplanar imaging system. For more details, see Bleich-Cohen et al. (2012).

### Imaging data analysis

Functional magnetic resonance imaging data were processed using the BrainVoyager 4.9 software package (http://www.brainvoyager.com). Preprocessing of functional scans included head movement assessment (scans with head movement >1.5 mm were rejected), high-frequency temporal filtering, and removal of low-frequency linear trends. To allow for T2* equilibration effects, the first six images of each functional scan were rejected. Pre-processed functional images were incorporated into the 3D datasets through trilinear interpolation. The complete dataset was transformed into Talairach space. Three-dimensional statistical parametric maps were calculated separately for each subject using a general linear model (GLM) in which the language condition as a predictor, using a lag of 3–6 s for an individual account of the hemodynamic response delay.

Specific effects were studied in a pre-determined region that is part of the language network (see below) and was defined individually based on commonly used anatomical landmarks, corresponding to Talairach coordinates [(L-IFG: *X* = −51, *Y* = 16, *Z* = 6); and reversed Talairach coordinates for the R-IFG: *X* = 52, *Y* = 15, *Z* = 6)] of activation clusters to the contrast of verb generation versus silence. We focused on the left IFG confined to BA 44–45 as representing Broca’s area and its homologous counterpart. Language-related activated clusters were obtained individually within the described anatomical landmarks (*p* < 0.01, uncorrected) using the GLM with the language condition as a positive predictor and the rest periods as negative predictors. The number of activated voxels in each of the defined ROIs on the left and right hemispheres was counted separately for each subject. These were used to compute a language-related lateralization index (LI) (i.e., LI = L−R/L+R, with L = number of voxels on the left and R = number of voxels on the right). The more positive the number, the more left lateralization was seen, while negative numbers represented lateralization to the right. Analysis of variance (ANOVA) was performed to explore group differences in lateralization using both the averaged LIs and number of active voxels in the ROI in each hemisphere using Statistica software (version 5.0).

### Functional connectivity analysis

Functional connectivity (FC) analysis was based on time-courses obtained from “seed region” located in the left IFG following a previously described procedure (Friston et al., 1993, 1995). The “seed region” was obtained from an individually defined activated cluster in the left IFG comprising of the most active 20-voxels in the contrast of language condition versus rest condition. These average time-courses were used as predictors in a GLM to compute a voxel-by-voxel fit. A second-level random-effect analysis and FDR of 0.0001 was applied to determine the brain areas that showed significant FC across subjects within each group. In order to quantify the inter-hemispheric effect, we extracted the *r* correlation coefficient from the voxels exhibiting peak of correlation in the right homolog IFG for each individual. We then looked for the correspondence between this measure and the value of LI for each individual within each group.

## Results

Table 1 presents demographic and clinical characteristics of the participants from the three patient groups.

**Table 1:** **Demographic and clinical characteristics of the study participants**.

Variable	Schizophrenia with OCD (*N* = 16)	Schizophrenia without OCD (*N* = 17)	OCD (*N* = 15)	Healthy controls (*N* = 20)
Age (years)	27.3 ± 3.4	25.7 ± 2.3	25.5 ± 3.5	26.4 ± 2.7
Gender (M/F)	10/6	11/6	11/4	12/8
Onset of schizophrenia (y)	19.9 ± 4.4	20.4 ± 3.4		
Duration of schizophrenia (y)	7.5 ± 4.6	6.2 ± 4.8		
Number of hospitalizations	1.3 ± 1.2	1.4 ± 2.1		
Onset of OCD (y)	15.5 ± 4.4	–	17.0 ± 2.4	
SAPS (total)	7.8 (3.6)	7.4 (3.9)		
SANS (total)	11.1 (4.1)	10.9 (4.6)		
CGI	4.1 (0.8)	4.3 (0.7)		
Y-BOCS (total)*	20.2 (8.2)	0.2 (0.7)	20.3 (5.7)	
Antipsychotic agents	16 patients	17 patients	3/15 patients	
Anti-obsessive agents**	8/16 patients	1/17 patients	14/15 patients	

*p < 0.001; **p < 0.01.

### Performance

Outside the scanner, all subjects performed the task with 100% accuracy. No difference was found between groups in reaction times (schizo-obsessive: 1.12 ± 0.3 s; SCH: 1.19 ± 0.3 s; OCD = 1 ± 0.1 s; controls: 0.9 ± 0.2 s, *F*(3, 37) = 1.88, *p* = 0.15, not shown). In addition, at the end of the fMRI scan, all subjects confirmed compliance during the task upon questioning.

## Imaging Results

### Hemispheric activation

**Figure 1:**
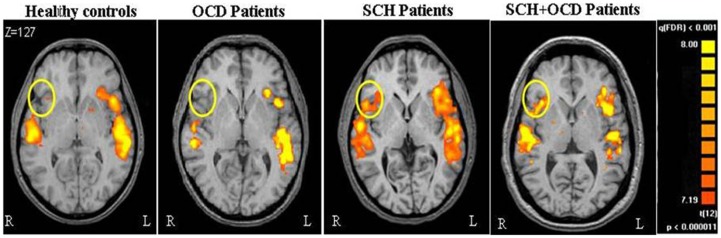
**Brain activation during language task is shown**. Axial views of parametric activation maps obtained during language tasks for the all groups. Colored regions indicate greater activation during VG than during periods of rest (FDR *p* < 0.001; random effect).

**Figure 2:**
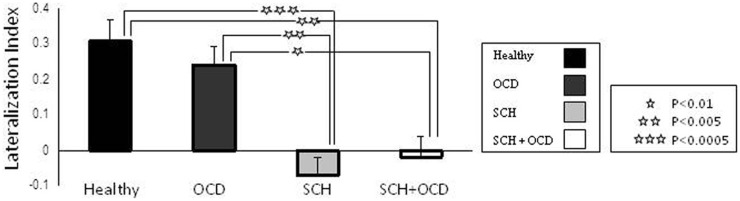
**Task-related activation per group and hemisphere is shown**. Lateralization index (LI) for language task across in the IFG. Error bars represent standard error of the mean (SEM). Stars indicate significant differences in activation between groups.

Figure 1 demonstrates the IFG lateralization in group activation maps obtained for the language task versus rest for all study groups. As expected, healthy controls and the OCD patients presented similar significant left lateralization, while the SCH group exhibited reduced lateralization for the language task. The schizo-obsessive group displayed reduced lateralization similar to the SCH group (LI measures: schizo-obsessive LI = −0.02; SCH patients LI = −0.07). In contrast, healthy controls and the OCD group demonstrated larger and more positive LIs (OCD patients LI = 0.24; controls LI = 0.3) (*F*(3, 48) = 11.69, *p* = 0.00, Figure 2). *Post hoc* analyses revealed significant differences between controls and SCH patients (Tukey HSD *post hoc*, *p* < 0.0005), controls and schizo-obsessive patients (Tukey HSD *post hoc*, *p* < 0.005), OCD patients and SCH patients (Tukey HSD *post hoc*, *p* < 0.005), and OCD patients and schizo-obsessive patients (Tukey HSD *post hoc*, *p* < 0.01), but no differences between SCH and schizo-obsessive patients. In order to further quantify this group difference, the number of activated voxels for language versus rest that was obtained for each hemisphere was used as a dependent variable in a two-way ANOVA (group and hemisphere as factors). A significant interaction was found showing greater asymmetry (i.e., left hemisphere dominance) for the controls and OCD patients than for the two groups of SCH patients [*F*(3, 48) = 10.28; *p* < 0.00]. *Post hoc* analyses revealed significant difference with increased amount of voxels in left versus right IFG of the controls and of the OCD patients (LSD *post hoc*, *p* < 0.0001, *p* < 0.005, respectively) and increased amount of voxels in right than left IFG of the two groups of SCH patients (LSD *post hoc*, *p* < 0.005,). In addition, there was a significant increase in the amount of voxels in the left IFG of healthy controls and OCD patients versus the SCH patients (LSD *post hoc*, *p* < 0.01, *p* < 0.05, respectively) and significant decrease in the right IFG of controls in comparison to SCH patients (LSD *post hoc*, *p* < 0.05) (not shown).

### Correlation map analysis

**Figure 3:**
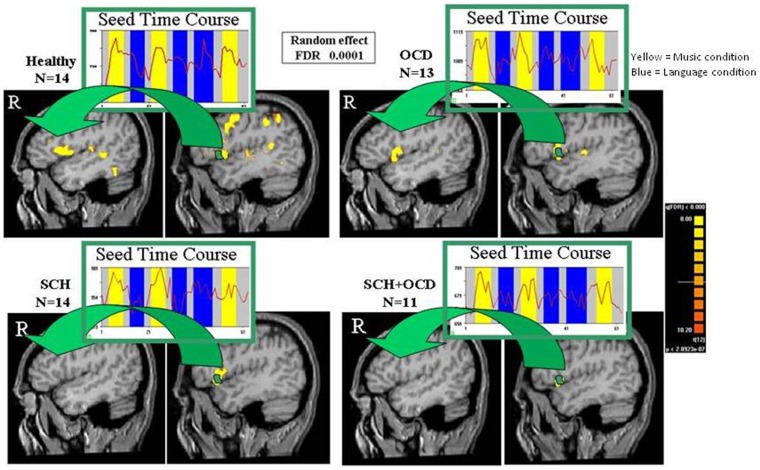
**Functional connectivity maps are shown**. Functional connectivity maps revealed by averaged time-courses from left IFG for vg vs. blank (random effect, FDR of 0.0001). Maps of functional connectivity are shown in sagittal views for all groups. The arrows point to the homolog IFG, where correlated activation with the left IFG was found for healthy controls and OCD, but not in SCH patients with and without OCD.

The response of the left IFG to the language condition was used as seed activation for a voxel-based functional correlation analysis per group. As depicted in Figure 3, schizo-obsessive patients presented no inter-hemispheric correlated activation as well as activation in the homolog IFG. This result was also obtained in the SCH patients. Contrarily, healthy controls and OCD patients demonstrated inter-hemispheric correlated activation as well as in the homolog IFG.

**Figure 4:**
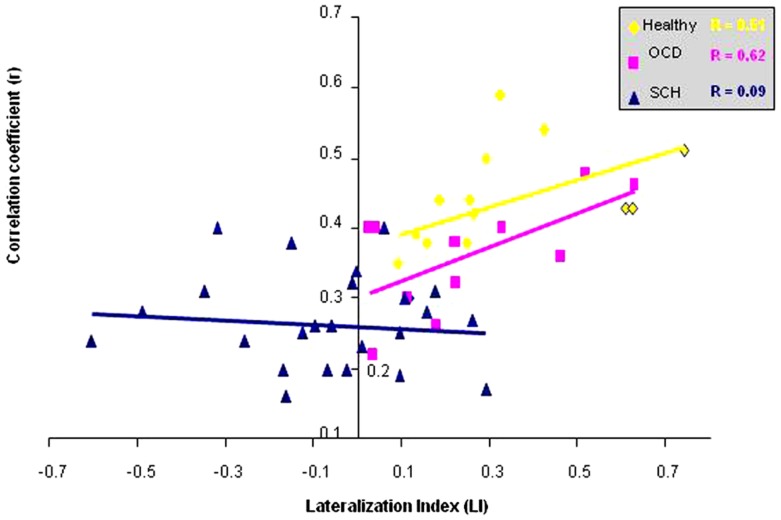
**Correlation between functional connectivity with left IFG and lateralization index is shown**. Correlation between the *r* correlation coefficient from right IFG and the LI for the healthy controls, OCD, and all SCH patients.

In the next stage, we calculated the *r* correlation coefficient of the peak voxel activation in the right IFG (conducted by the FC analysis with the left IFG taken as a seed region) for each subject and group values for controls, OCD patients, and all SCH patients separately. We conducted correlation analysis between the *r* correlation coefficient of right IFG and LI values for all the groups. In addition, we conducted correlation analysis between the *r* correlation coefficient of the right IFG and (1) number of activated voxels in the right IFG and (2) symptom severity scores for all SCH patients. We found three significant results. In the correlation analysis between the *r* correlation coefficient of the right IFG and LI values, healthy controls and OCD presented significant correlation (*r* = 0.51, *p* = 0.06; *r* = 0.62, *p* = 0.02, respectively), while SCH patients did not (Figure 4).

We also found a negative correlation between language lateralization and positive symptoms (*r* = 0.4), which was close to significance (*p* = 0.06). In addition, a negative correlation was obtained between the *r* correlation coefficient and the number of activated voxels in the right Broca (*r* = 0.44), which was significant (*p* = 0.03) in all SCH patients. Furthermore, negative correlation was obtained between the *r* correlation coefficient and the average score of negative symptoms (including affect, avolition, anhedonia, and alogia) (*r* = 0.39), which was close to significance (*p* = 0.06) in all SCH patients.

## Discussion

The aim of the current fMRI study was to examine whether neural mechanisms involved in language processing differ in SCH patients with OCD when compared to SCH patients with no co-morbidity. Specifically, following our previous findings of abnormal language lateralization in patients with SCH but not in OCD (Bleich-Cohen et al., 2012), we sought to examine whether the presence of OCD can attenuate abnormal language processing in co-morbid schizo-obsessive patients, acting as a putative “protective” mechanism.

Contrary to our assumption, while performing the language task, the two SCH groups with and without OCD revealed similar patterns of brain activation, namely reduced lateralization in the IFG and diminished inter-hemispheric FC. This may be explained by the significant negative correlation that we found between inter-hemispheric connectivity and the amount of activated voxels in the right IFG in all SCH patients. However, it is also consistent with the current notion that large scale neural network abnormalities and a widespread “Disconnection Syndrome” underlie this archetypal psychotic illness. The “Disconnection Syndrome,” which is a leading theory in SCH research, claims that disturbed long-distance connections between frontal and posterior brain regions encompasses the core neuropathology in SCH (Friston et al., 1993; Andreasen et al., 1999). This idea is supported by imaging studies showing abnormal fronto-temporal FC in SCH (Wright et al., 2000; Bleich-Cohen et al., 2009b). The current study further suggests that in addition to reduced connectivity within the hemispheres, there is possibly a disrupted connection between the hemispheres.

In addition, our findings of a negative correlation between inter-hemispheric connectivity and symptoms, similar to Sommer et al. (2001) who demonstrated negative correlation between the degree of lateralization and the severity of hallucinations, further implicate such widespread disconnectivity in the pathophysiology and phenomenology of SCH.

Our findings suggest that regardless of the presence of OCD, SCH patients fail to show brain lateralization characteristic of normal language processing. Contrary to SCH in which perturbed verbal fluency and a poor grasp of semantics are core features, language function is generally preserved in OCD (Millan et al., 2012). Preserved IFG function and FC in OCD is therefore consistent with the leading model implicating fronto-striatal (and especially OFC and ventromedial PFC) circuit dysfunction that spares the IFG in OCD, although it is emerging that there may be a more widespread network involvement in OCD than previously assumed (Hendler et al., 2014). This, however, seems not to be translated into more efficient language-related brain processing in those SCH patients who exhibit also OCD. Notably, a similar pattern of lack of a modifying effect of co-morbid OCD on brain activation in SCH patients has been found when the N-back working memory task was employed (Bleich-Cohen et al., 2014). In this study, the two SCH groups with and without OCD failed to adequately recruit a working memory network when facing increasing working memory load, exhibiting a similar reduction in activation in the right dorso-lateral PFC and right caudate, as well as decreased FC compared to the healthy controls.

Overall, the use of tasks tapping cognitive domains, which have consistently been implicated in cognitive dysfunction in SCH (e.g., working memory, language processing) seems not to discriminate between SCH subgroups with and without OCD. Thus, the use of other tasks that more explicitly address the OCD component in co-morbid schizo-obsessive patients (e.g., reinforced learning, response inhibition) may potentially differentiate between brain activation patterns in SCH patients with and without OCD.

Several limitations of the present study should be noted. All SCH patients received antipsychotic agents, which could have affected patterns of brain activation, limiting the generalization of our findings. Moreover, significantly more patients in the schizo-obsessive group were treated with add-on SSRIs to address symptoms of OCD. However, the effect of this treatment on brain activity is yet to be clarified. An additional limitation of the current study is the lack of condition separation in the FC analysis.

## Conflict of Interest Statement

The authors declare that the research was conducted in the absence of any commercial or financial relationships that could be construed as a potential conflict of interest.
